# Surveying soil-borne disease development on wild rocket salad crop by proximal sensing based on high-resolution hyperspectral features

**DOI:** 10.1038/s41598-022-08969-5

**Published:** 2022-03-24

**Authors:** Angelica Galieni, Nicola Nicastro, Alfonso Pentangelo, Cristiano Platani, Teodoro Cardi, Catello Pane

**Affiliations:** 1 Council for Agricultural Research and Economics (CREA), Research Centre for Vegetable and Ornamental Crops, via Salaria 1, 63077 Monsampolo del Tronto, Italy; 2 Council for Agricultural Research and Economics (CREA), Research Centre for Vegetable and Ornamental Crops, via Cavalleggeri 25, 84098 Pontecagnano Faiano, Italy; 3 Present Address: CNR-IBBR, Institute of Biosciences and BioResources, via Università 133, 80055 Portici, Italy

**Keywords:** Plant sciences, Optics and photonics

## Abstract

Wild rocket (*Diplotaxis tenuifolia*, Brassicaceae) is a baby-leaf vegetable crop of high economic interest, used in ready-to-eat minimally processed salads, with an appreciated taste and nutraceutical features. Disease management is key to achieving the sustainability of the entire production chain in intensive systems, where synthetic fungicides are limited or not permitted. In this context, soil-borne pathologies, much feared by growers, are becoming a real emergency. Digital screening of green beds can be implemented in order to optimize the use of sustainable means. The current study used a high-resolution hyperspectral array (spectroscopy at 350–2500 nm) to attempt to follow the progression of symptoms of Rhizoctonia, Sclerotinia, and Sclerotium disease across four different severity levels. A Random Forest machine learning model reduced dimensions of the training big dataset allowing to compute de novo vegetation indices specifically informative about canopy decay caused by all basal pathogenic attacks. Their transferability was also tested on the canopy dataset, which was useful for assessing the health status of wild rocket plants. Indeed, the progression of symptoms associated with soil-borne pathogens is closely related to the reduction of leaf absorbance of the canopy in certain ranges of visible and shortwave infrared spectral regions sensitive to reduction of chlorophyll and other pigments as well as to modifications of water content and turgor.

## Introduction

Wild rocket (*Diplotaxis tenuifolia* [L.] D.C.) is a leafy vegetable crop belonging to the *Brassicaceae* family, cultivated in many temperate areas worldwide for fresh consumption in salad preparations, and appreciated for the characteristic pungency, intense peppery flavour, antioxidant activity and other health-promoting phytonutrients^1–3^. This baby-leaf crop has a significant economic interest particularly with reference to the specific high-convenience food chain^4^. Nowadays, Italy is the major European producing country of packaged wild rocket, cultivated on around 6000 ha under unheated plastic greenhouses^5,6^. Because of intensive farming, multiple harvesting cycles *per* sowing season at 20–40 days intervals^7^, specific protocols, based on low-impacting agrotechniques, are necessary with the crucial aim to avoid pesticide residues in the product and obtain premium quality fresh-cuttings associated with improved aesthetic look, taste, nutraceutical properties and shelf-life potential^8–10^. Nevertheless, the growing conditions, general cultivar susceptibility to pathogens and the requested lower use of fungicides make the crop prone to recrudescence and/or re-emergence of soil-borne pathologies^11–13^, such as *Rhizoctonia solani* Kühn and *Sclerotinia sclerotiorum* (Lib.) de Bary, which can cause devastating lawn rotting and yield losses impacting the farmer economy in the short and mid-term^11,14,15^. These soil-borne pathologies rise to a real emergency in the intensive biological management systems and anywhere synthetic fungicides are banned or not permitted^16–20^. Valuable alternatives appliable in the management programs, need to be further improved in terms of use efficiency^21^. Digital technologies that analyse the light reflected from crop foliage in a wide wavelength range of the electromagnetic spectrum can aid in the making decision process aiming to crop protection targeted strategies based on a reduced use of pesticides^22,23^. For example, Manganiello et al.^24^ recently selected high-performing hyperspectral vegetative indices (VIs), such as Soil Adjusted Vegetation Index (SAVI) group and Triangular Vegetation Index (TVI), able to track *Trichoderma* spp. biocontrol strains in counteracting multiple soil borne diseases of baby leaf vegetables, including *R. solani* on wild rocket, opening the way to precision biological control applications.

The non-imaging technology is based on the application of high-resolved reflectance spectroscopy to acquire the punctual spectral fingerprints of a plant at foliar and/or canopy level, interrelated to its chemical-physical attributes, in order to provide useful remote sensed information on the plant status. Previous studies demonstrated the accuracy of hyperspectral optical sensing to well classified plant diseases and configure reference outlines for the proximal surveying of crop healthiness^25^. Karadağ et al.^26^ improved a method based on 350–2500 nm spectral reflectance for the *Fusarium* disease severity estimation on pepper. On peanut, reflectance data have been used to identify the disease stage of the bacterial wilting caused by *Ralstonia solanacearum*^27^. Junges et al.^28^ applied hyperspectral scouting to discriminate the described symptomatology associated with the grapevine decline and death syndrome. In tomato, following a laboratory-to-field approach, Abdulridha et al.^29^ pointed up hyperspectral surveying cultivations affected by non-specific leaf spotting at different stages, caused by the fungus *Corynespora cassicola* and the bacterium *Xanthomonas perforans*.

The above-mentioned technologies offer new opportunities to devise innovative tools for supporting the sustainable soil-borne disease management of leafy vegetables, increasing the farmers' monitoring capability to early identify outbreaks and apply a timely and spatially effective phytosanitary intervention, especially under continuous sequences in Mediterranean-type climate regions^30^. However, a basic computational study to filter the acquiring big data into more manageable synthetic hyperspectral indices is a decisive step to make the proposed digital tool exploitable in practical applications on crops with acceptable costs^31^. Therefore, this study aimed at the evaluation of the disease-specific hyperspectral signatures of wild rocket plants artificially inoculated, under laboratory conditions, with three soil-borne pathogens (*R. solani*, *S. sclerotiorum* and *Sclerotium rolfsii* Sacc.). To this purpose, wild rocket plants were subjected to hyperspectral data acquisition using a non-imaging hyperspectral sensor. The first objective was to obtain a high-resolution assessment of the differences among spectral signatures of healthy and infected (at different disease levels) plants at leaf scale, and thus the selection of the most discriminant wavelengths. This information was used to set specific de novo synthetic VIs under the most stable environmental conditions, without risk of changing environmental factors (i.e., using the sensor as an active sensor), through a Machine Learning pipeline using the Random Forest (RF) algorithm. The second objective was to understand factors affecting the use of newly developed VIs at plant/canopy scale (i.e., at pot scale and using the sensor as passive sensor), assessing their transferability to high-throughput experiments for practical applications. The methodology devised in the present study can be of larger applicability in other crop—(soil-borne) pathogen systems.

## Materials and methods

The present study was conceptualized and conducted based on two independent experiments, named “guide experiment” (Exp_Gui) and “applicative experiment” (Exp_App), following the workflow shown in Fig. S1.

Leaf and canopy reflectance data (see the “Reflectance measurements” section) were collected from soil-inoculated wild rocket plants grown in controlled environments. From Exp_Gui we obtained leaf-scale reflectance data. The resulting dataset was used for calibration and validation purposes, and then divided into a “training dataset” and a “testing dataset” (see the “Data preparation and analysis” section), to model spectral responses at increasing levels of disease severity, and to develop new VIs capable of monitoring aerial symptoms of soil-borne diseases in wild rocket. Reflectance data at canopy scale were acquired from Exp_App. The resulting dataset, called the “canopy dataset”, was used to evaluate the applicability of the newly developed VIs using plant growth and data acquisition conditions comparable to those normally applicable, or expected to be applicable, in real growth environments for early detection of fungal diseases.

### Fungal pathogens and inoculum preparation

Virulent isolates of soil-borne fungal pathogens *R. solani* (RhS) AG-4, *S. rolfsii* (ScR), and *S. sclerotiorum* (ScS) used in this study were obtained from the collection of fungi maintained on potato dextrose agar (PDA, Difco Laboratories, Detroit, Mich.) slants at 20 °C at the Research Centre for Vegetable and Ornamental Crops, Council for Agricultural Research and Economics (CREA), located in Pontecagnano Faiano, Italy. They were previously tested for pathogenicity on wild rocket; stock cultures were stored on PDA slants at 4 °C until use. For each pathogen, the artificial inoculum was prepared by infecting with 20 mycelia plugs (0.5 cm diameter) 500 g millet, previously autoclaved (22 min., 121 °C) and saturated (v/v) with 0.1 × potato dextrose broth (PDB, Difco Laboratories, Detroit, Mich.). After 21 days of incubation in the dark at 25 °C, the infected millet was ready for use after air-drying.

### Experiment description

In both experiments, wild rocket (*D. tenuifolia*) cv. Tricia (Enza Zaden, Italy) was sown in black plastic pots (140 mm diameter) filled with autoclaved peat substrate (Klasmann-Deilmann GmbH, Geeste, Germany), at a density of 5 plants pot^−1^, and grown in a nursery glasshouse to obtain 1-month-old plants. Then, plants were transferred to benches in a climatic chamber at 25 ± 2 °C with a 12-h photoperiod (Exp_Gui) or in an experimental greenhouse (Exp_App). Daily irrigation with dechlorinated sterile water was performed to maintain water holding capacity around 80% of nominal, while a basal NPK mix liquid fertilization was applied twice a week.

For both Exp_Gui and Exp_App, three disease conditions (RhS-D, ScR-D, and ScS-D, disease) were imposed, each at three different levels of disease severity plus a healthy not-inoculated treatment. Each treatment consisted of a total of 10 pots (replicates) for each combination. The different levels of disease severity were obtained by inoculating different groups of plants at 10, 7, and 3 days before sampling, i.e., spectral acquisitions. Pots were inoculated by incorporating 1 g (dry weight) pot^−1^ of infected millet in the first layer (5 cm depth) of the substrate. Eventually, each pathosystem consisted of 40 pots (200 plants), characterized by different levels of disease severity for a total of 120 pots (600 plants) in the whole trial.

**Figure 1:**
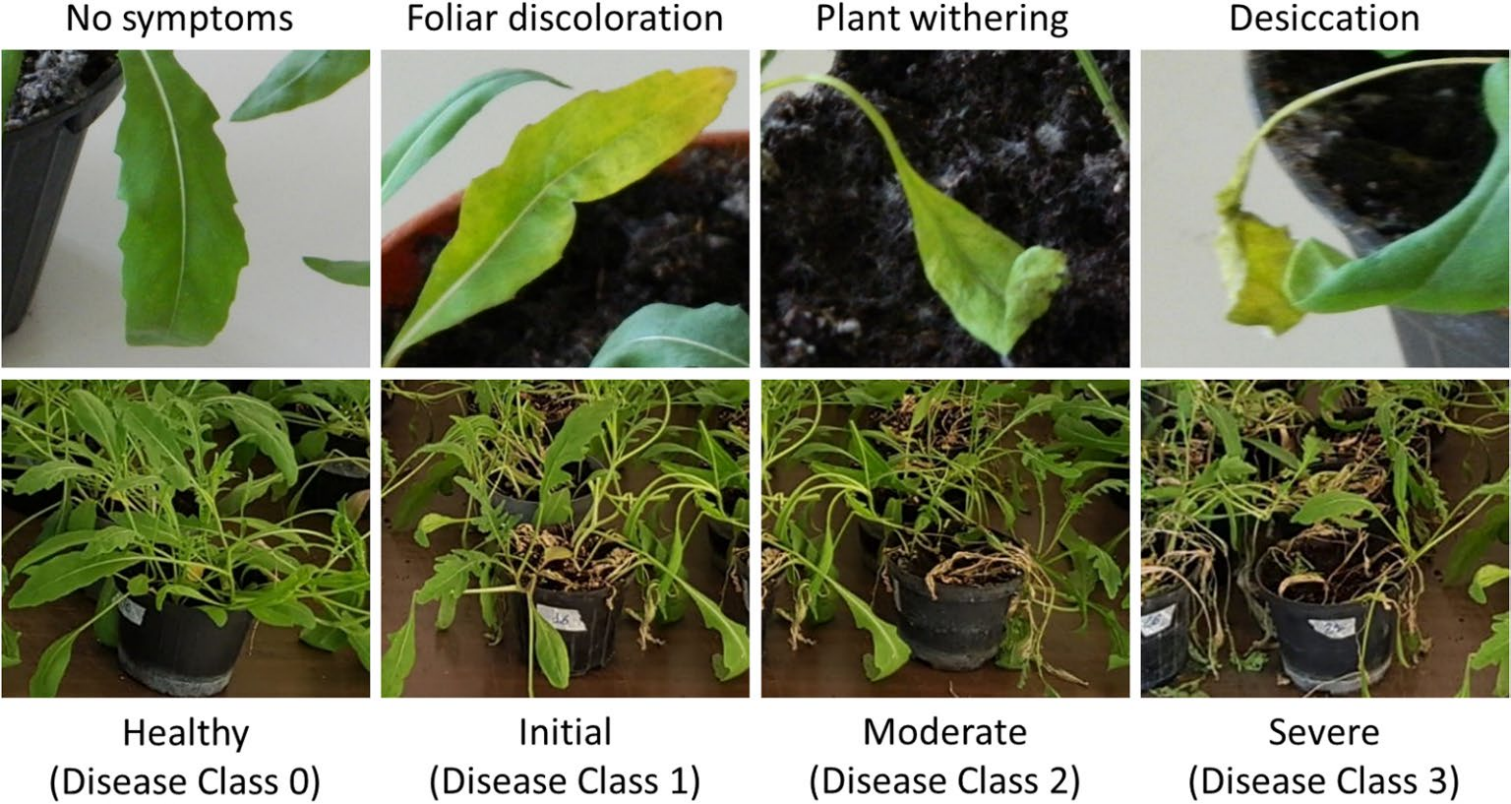
Representative photographs of the four disease classes (0–3) at foliar and pot level.

Leaves from Exp_Gui were visually assessed for their disease severity with a score on a 0–3 scale. For pots from Exp_App a visual inspection was also assessed by using a 0–3 rating scale according to the mean symptoms of foliage disease, for each pathosystem. Leaves/pots were classified as: healthy (Disease class 0), and diseased at early (Disease class 1), moderate (Disease class 2), and severe (Disease class 3) degree, according to Manganiello et al.^24^. Disease class 0 was observed only from the not inoculated healthy controls (Fig. 1).

### Reflectance measurements

In Exp_Gui, leaf reflectance (350–2500 nm) was measured by contact under laboratory conditions with a portable non-imaging spectroradiometer (FieldSpec^®^ 4 Hi-Res, ASD Inc., Boulder, CO, USA) using an optical fiber contact probe (ASD Plant Probe; ASD Inc., USA) with a 10 mm field of view and an integrated halogen reflector lamp, which minimized atmospheric interference on the recorded signal (active sensor). To increase the quality and homogeneity of the acquired data, the instrument was warmed up for 90 min before measurement. The pre-calibrated Spectral 99% white reference panel was used for calibration, run every 5 min during data acquisition. Each sample scan represented an average of 20 reflectance spectra. Leaves were randomly selected from pots/plants characterized by different disease levels, resulting in a homogenous sampling across disease severity classes. A total of 216, 212, and 222 reflectance acquisitions were obtained for RhS-D, ScR-D and ScS-D, respectively. Leaves were removed from the plants and spectral reflectance was measured immediately on-site on their adaxial side.

For Exp_App, the canopy reflectance was assessed outdoors under full sunlight conditions using the ASD FieldSpec^®^ 4 Hi-Res (ASD Inc., Boulder, CO, USA). All spectra were obtained using the spectroradiometer with a 25° field of view (FOV). Potted plants were placed in a fixed plate on a black background, maintaining a vertical distance between the sensor and the top of the plants of 10 cm (passive sensor). Three measurements *per* pot were conducted to obtain an average spectrum in the “canopy dataset” (n = 40 for each disease).

### Dry weight, leaf area index

Plants in Exp_App were also evaluated for dry weight (DW, g pot^−1^), leaf area (LA, cm^2^ pot^−1^), and dry matter content (DM, %) of aerial biomass. Plants were sampled and transferred to the laboratory for aerial fresh and DW determinations, after drying them in an oven at 70 °C until constant weight. Prior to drying, LA was recorded using LI-3100 area meter (Li-Cor Corporation, Lincoln, NE, USA).

### Data preparation and analysis

After removing the noisy bands at the extreme wavelengths, the spectral range from 400 to 2450 nm (2051 wavelengths) was analysed. For both leaf and canopy reflectance data, pre-processing involved splice correction to remove the abrupt changes in the recorded values of the neighbouring wavelengths, which appear at the spectral intervals between the three integrated sensors of ASD FieldSpec spectroradiometer. Splice correction was achieved using the commercial software package ASD-View Spec Pro (ASD Inc., Boulder, CO, USA).

Leaf reflectance was assessed under constant light and temperature conditions, so further pre-processing to smooth the spectrum and reduce signal noise was not performed^32^. Prior to spectral statistical analyses, leaf reflectance was pre-processed using first derivative and mean-centering, in order to reduce the baseline offset by amplifying small spectral features^33^. Data pre-treatments was performed with the ParLeS software^34^. For each disease, data were then randomly divided into two datasets by splitting the observations (approximately 70:30) into training (calibration) and test (validation) datasets (training set, n = 150, 149, and 160 for RhS-D, ScR-D and ScS-D, respectively; testing set, n = 66, 63, and 62 for RhS-D, ScR-D and ScS-D, respectively).

The RF supervised machine learning algorithm^35^, a classification algorithm consisting of an ensemble of decision trees, was used by applying R Caret package^36^ to select the most predictive bands to be used in the construction of the VIs as well as to evaluate the VIs performance in discriminating among the four classes for each disease. To this purpose, a two-step strategy was applied using two types of continuous variables, i.e., (i) reflectance features and (ii) VIs derived from Exp_Gui, to classify the categorical variables (Disease Classes 0, 1, 2, 3) through a set of decision tree process to develop the RF predictive models. The complexity of the input variables was then reduced gradually by selecting at each step the features (bands or VIs) with the highest predictive power through the VSURF R package^37^ and testing the performance of the restricted variable sets using the Confusion Matrix (R Caret package) and Kappa coefficient. In total, 4 RF models were obtained using: for the first step, (1) all the wavelengths (All λ) and (2) only the selected wavelengths (Selected λ); for the second step, (3) all the derived VIs (All VIs) and (4) only the selected VIs (Selected VIs).

The VIs related to disease severity classes were calculated from the selected wavelengths in the most important regions of the full spectrum. For RhS-D, ScR-D and ScS-D, wavelength selection was performed during the first RF step.

Two band combinations of the spectral indices, Normalized Difference Spectral Indices (NVIs) and Simple Ratio Indices (SRs) were generated for all the possible combinations of the selected wavelengths following the equations:

$${NVI}_{i-j}=\frac{(reflectance({wavelength}_{i})-reflectance({wavelength}_{j}))}{(reflectance({wavelength}_{i})+reflectance({wavelength}_{j}))}$$

$${SR}_{i-j}=\frac{reflectance({wavelength}_{i})}{reflectance({wavelength}_{j})}$$

where *reflectance(wavelength*_*i*_*)* and *reflectance(wavelength*_*j*_*)* are the spectral reflectance values of two random wavelengths (λ*i* and λ*j*, respectively).

The newly obtained NVIs and SRs were correlated (Spearman rank correlation)^38^ with the disease severity levels for each pathogen, to identify the most suitable indicators associated with the identification and development of the pathogenesis.

Finally, newly developed VIs strongly correlated with treatments were tested using the canopy dataset, obtained by canopy reflectance measurements from Exp_App. For each disease, Spearman rank correlations were applied with R software^39^ to assess the correlation between the VIs and disease severity (at the pot scale) as well as between the VIs and LA, DW and DM, all performed at the pot scale. NVIs and SRs were calculated from the raw spectra after splice correction, as previously indicated. One-way ANOVA followed by the post hoc Least Significant Difference test (LSD) was applied to assess statistical relationships among VIs values over the disease classes using the R package agricolae^40^.

Machine learning modelling, correlations and ANOVA were performed using the statistical software R version 4.0.2^39^.

### Ethics approval

The experimental research and field studies on plants, including the collection of plant material, complied with relevant institutional, national, and international guidelines and legislation.

## Results

### Disease index and plant growth

All plants in the infected pots developed disease symptoms, as expected, with varying severity depending on the specific pathogen and timing of inoculation. ScR proved to be the most aggressive pathogen producing the basal hydropic lesions immediately after the first day of interaction. Overall, all diseases progressed from hydropic halos/lesions on the plant collar and basal parts of the petioles to rot, wilt, and death of the plant. ScR formed the typical mycelial felt on the targeted basal organs and at a late stage produced the typical light brown spherical sclerotia. On the other hand, ScS signalled its presence with a slight production of white mould, whereas RhS did not burst.

**Figure 2:**
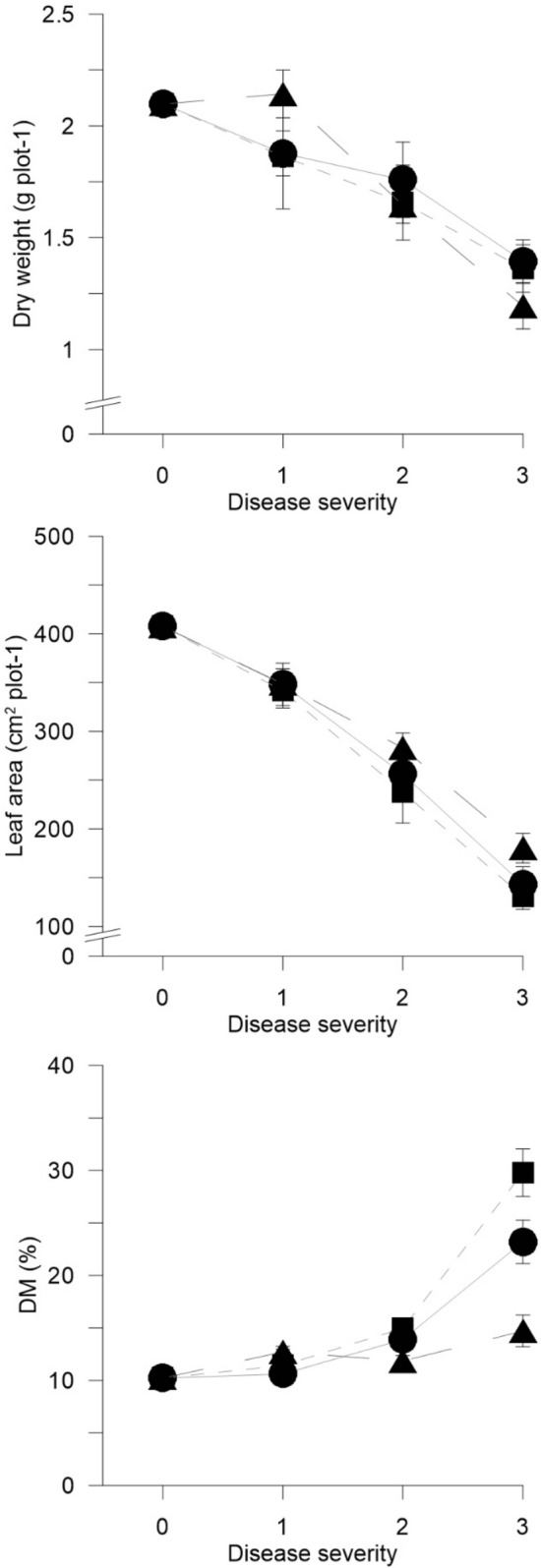
Dry weight (DW, g plot^-1^), leaf area (LA, cm^2^ plot^-1^), and dry matter (DM, %) as recorded for wild rocket inoculated with *Rhizoctonia solani* (RhS-D, black circle), *Sclerotium rolfsii* (ScR-D, black square), and *Sclerotinia sclerotiorum* (ScS-D, black triangle), at four disease severity levels (Disease classes 0, 1, 2 and 3 – x-axis) (Exp_App). Data are averages ± standard errors.

Based on Exp_App, the effects of soil-borne pathogens on plant growth were monitored. Fungal diseases significantly affected plant DW, LA and DM, as they progressed through the four severity levels (Fig. 2).

### Hyperspectral fingerprints

**Figure 3:**
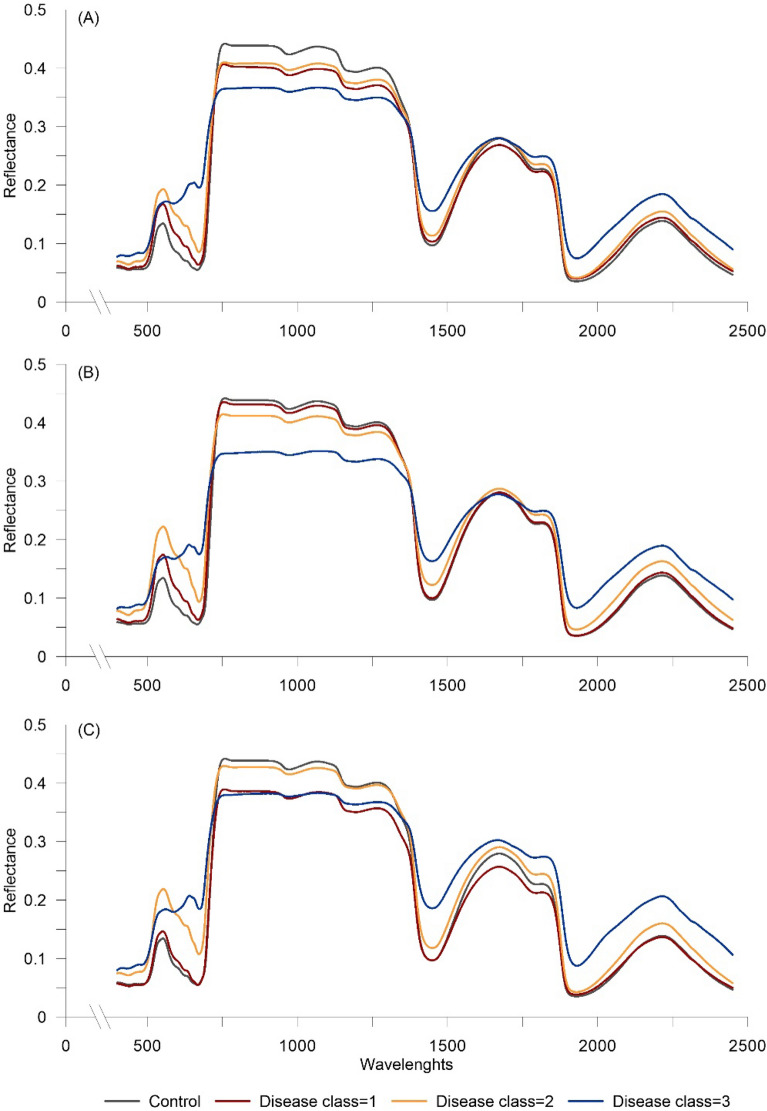
Spectral reflectance as recorded, in the range 350-2500 nm, on wild rocket leaves inoculated with (**A**) *Rhizoctonia solani* (RhS-D), (**B**) *Sclerotium rolfsii* (ScR-D), and (**C**) *Sclerotinia sclerotiorum* (ScS-D) at four disease severity levels (Disease classes 0, 1, 2 and 3) (Exp_Gui).

Typical reflectance signatures of diseased leaves of wild rocket inoculated with RhS, ScR, and ScS are shown in Fig. 3A–C, respectively. Regardless of the fungal pathogen, the reflectance of leaves of infected plants tended to increase abruptly in the VIS region with advancing disease severity. At the highest disease severity level, the profile of the spectral signature was also affected, especially between 550 and 700 nm, resulting in the altered redshift characteristics and red-edge positions. Despite differences among the pathogens, the reflectance of the most infected leaves decreased in the NIR regions and increased again from around 1400 nm, without affecting the signature patterns (Fig. 3).

### Selection of the features by Random Forest modelling

In order to select the most discriminant wavelengths for each pathogen, the RF supervised machine learning algorithm was trained and validated using, respectively, 70 and 30% of the input observations from Exp_Gui. The predictive performance of the models (All λ) is highlighted in each related Caret's Confusion Matrix and related statistics shown in Table 1. RF ran efficiently on the first derivative reflectance data showing a stable prediction of disease levels compared to the healthy Control, already at the early stages of infection. The higher accuracy level was recorded for ScR-D (0.726) followed by RhS-D and ScS-D (0.625, and 0.500, respectively); these accuracy levels were significantly greater than the no information rate (*p*-value [A > N] < 0.05).

**Table 1:** Confusion Matrices and overall statistics of Random Forest models trained and validated on (1) all wavebands (All λ) and on (2) the selected wavebands (Selected λ)—reflectance data recorded from wild rocket leaves inoculated with *Rhizoctonia solani* (RhS-D), *Sclerotium rolfsii* (ScR-D), and *Sclerotinia sclerotiorum* (ScS-D) at four disease severities (Disease classes 0, 1, 2 and 3) (Exp_Gui).

		RhS-D						ScR-D					ScS-D			
			True classes					True classes					True classes			
All λ	*Predicted classes*		0	1	2	3		0	1	2	3		0	1	2	3
		0	12	9	3	0	0	16	4	2	0	0	14	9	4	1
		1	2	10	8	0	1	2	11	5	0	1	2	3	1	1
		2	0	1	4	1	2	0	2	8	0	2	1	6	6	4
		3	0	0	0	14	3	0	1	1	10	3	0	2	2	10
Accuracy				0.625					0.726					0.500		
95% confidence interval				(0.4951, 0.7430)					(0.5977, 0.8315)					(0.3743, 0.6257)		
No information rate				0.3125					0.2903					0.303		
*p*-value [A > N]				2.621 e^−07^					1.725 e^−12^					6.313 e^−04^		
Kappa				0.4980					0.6294					0.3390		
Selected λ	*Predicted classes*		0	1	2	3		0	1	2	3		0	1	2	3
		0	19	7	1	1	0	17	6	3	0	0	15	9	4	2
		1	1	11	2	0	1	1	9	5	0	1	1	4	1	0
		2	0	1	10	1	2	0	3	7	2	2	1	6	7	4
		3	0	0	1	9	3	0	0	1	8	3	0	1	1	10
Accuracy				0.762					0.661					0.546		
95% confidence interval				(0.6379, 0.8602)					(0.5299, 0.7767)					(0.4181, 0.6686)		
No information rate				0.3016					0.2903					0.3030		
*p*-value [A > N]				6.623 e^−14^					1.633 e^−09^					3.578 e^−05^		
Kappa				0.6755					0.5390					0.4000		

Wavelenghts were selected by the recursive elimination of the smallest important ones to obtain a subset of explanatory variables highly related to the model response.

The R package VSURF was then implemented on the obtained RF models in order to remove irrelevant information by the recursive elimination of the smallest important wavelengths and so leading to a subset of explanatory variables highly related to the model response, by minimizing the prediction error. This variable selection method returns a smaller subset of variables focusing more closely on the RF prediction objective avoiding redundancy. The second RF model (Selected λ) was then constructed for each pathogen. In this case, we observed high levels of the proportion of correctly classified cases as well as good metrics, indicating substantial invariance in the predictive value of the models compared to the previous benchmark and so demonstrating the efficiency of band selection (Table 1). In particular, an increase of accuracies of RF models for RhS-D and ScS-D (0.762 and 0.546, respectively), and a decrease for the ScR-D ones, were observed. Moreover, the Balanced Accuracy predicted better the two extreme disease classes (Class 0 and 3)—with values > 0.75—than the intermediate ones (Class 1 and 2), both when all wavelengths were used as entry variables of models and when the RF models were constructed using only the selected wavelengths (Tables S1, S2, S3). This also emerges from the confusion matrices where the misclassified observations tend to zero as we move away from the diagonal (Table 1).

For each telluric pathogenic disease, the selected wavelengths from RF models, most related to the classification response, are reported in Table 2. These bands fell in three regions of the full spectrum, mainly in the VIS–NIR and very few in the SWIR. A total of 9, 16, and 12 wavelengths for RhS-D, ScR-D, and ScS-D, respectively, were selected and combined to build 135, 72, 99 both SRs and NVIs, respectively, for each disease (data not shown). It is worth noting that, regardless of the pathogen, the models selected neighbouring wavelengths in the spectrum (e.g., 559/560 nm; 601/602 nm; around 665 nm; around 724 nm). Some differences were observed for ScR-D in the VIS (i.e., 447 and 854 nm) and, in general among diseases, in the SWIR region.

**Table 2:** Wavelengths (nm) selected from reflectance data recorded on wild rocket leaves inoculated with *Rhizoctonia solani* (RhS-D), *Sclerotium rolfsii* (ScR-D), and *Sclerotinia sclerotiorum* (ScS-D) (Exp_Gui).

Disease	VIS–NIR	SWIR
RhS-D	559-602-643-664-671-703-719-724	1377
ScR-D	447-560-567-579-602-636-658-662-663-667-684-724-733-735-854	1958
ScS-D	560-575-601-656-667-719-721-725-726	1135-1905-1908

The wavelenghts were selected starting from RF models by the recursive elimination of the smallest important ones. VIS–NIR = visible-near infrared regions; SWIR = short-wave infrared region.

The so obtained VIs were used for the construction of the third RF classification model (All VIs) for each disease. Indeed, thanks to the flexibility of the RF algorithm, all VIs can be used to get a more accurate prediction of the RhS-D, ScR-D and ScS-D levels. The related confusion matrix showed general accuracy predictions of all VIs based RF models that stood at 0.656, 0.645, and 0.530 for RhS-D, ScR-D, and ScS-D, respectively (Table 3).

**Table 3:** Confusion Matrices and overall statistics of Random Forest models trained and validated on (3) all the VIs generated by the combinations of the selected wavelengths (All VIs) and on (4) the selected VIs (selected VIs)—reflectance data recorded from wild rocket leaves inoculated with *Rhizoctonia solani* (RhS-D), *Sclerotium rolfsii* (ScR-D), and *Sclerotinia sclerotiorum* (ScS-D) at four disease severities (Disease class 0, 1, 2 and 3) (Exp_Gui).

		RhS-D						ScR-D					ScS-D			
			True classes					True classes					True classes			
All VIs	Predicted classes		0	1	2	3		0	1	2	3		0	1	2	3
		0	13	12	2	0	0	15	8	3	0	0	13	8	3	1
		1	1	7	2	1	1	3	8	4	0	1	4	5	2	1
		2	0	1	9	1	2	0	2	7	0	2	0	5	8	5
		3	0	0	2	13	3	0	0	2	10	3	0	2	0	9
Accuracy				0.656					0.645					0.530		
95% confidence interval				(0.5270, 0.7705)					(0.5134, 0.7626)					(0.4034, 0.6544)		
No information rate				0.3125					0.2903					0.3125		
*p*-value [A > N]				1.664 e^−08^					7.577 e^−09^					2.621 e^−07^		
Kappa				0.5470					0.5201					0.3764		
Selected VIs	*Predicted classes*		0	1	2	3		0	1	2	3		0	1	2	3
		0	10	8	2	0	0	14	6	1	0	0	14	8	3	1
		1	3	7	5	1	1	4	9	6	0	1	3	5	2	0
		2	1	5	5	3	2	0	3	7	2	2	0	5	7	4
		3	0	0	3	11	3	0	0	2	8	3	0	1	1	11
Accuracy				0.516					0.613					0.561		
95% confidence interval				(0.3873, 0.6425)					(0.4807, 0.7340)					(0.4330, 0.6826)		
No information rate				0.3125					0.2903					0.3030		
*p*-value [A > N]				5.709 e^−04^					1.332 e^−07^					1.225 e^−05^		
Kappa				0.3550					0.4746					0.4166		

VIs were selected by the recursive elimination of the less important indices to obtain a subset of explanatory variables highly related to the model response.

These models were propaedeutic for the most effective classifier VIs selection; indeed, the subsequent VSURF step allowed the selection of the 12, 10, and 10 most suitable indices, listed in Table 4 and described in the next section, to model disease severity for their ability to classify via RF algorithm the RhS-D, ScR-D and ScS-D. To confirm their consistency for the mentioned objective, the newly obtained VIs fed the fourth RF model (Selected VIs), giving metrics shown in Table 3 as well as in Tables S1, S2, and S3.

**Table 4:** Hyperspectral vegetation normalized difference (NVI) simple ratio (SR) indices (dimensionless) used in this study for wild rocket disease detection.

Index	Formula	Reference pathogen	Spearman’s rank correlation coefficient
NVI_559-602_	(R_559_-R_602_)/(R_559_ + R_602_)	RhS	− 0.788**
NVI_559-671_	(R_559_-R_671_)/(R_559_ + R_671_)	RhS	− 0.382**
NVI_602-559_	(R_602_-R_559_)/(R_602_ + R_559_)	RhS	0.788**
NVI_643-559_	(R_643_-R_559_)/(R_643_ + R_559_)	RhS	0.692**
NVI_703-724_	(R_703_-R_724_)/(R_703_ + R_724_)	RhS	0.741**
NVI_724-602_	(R_724_-R_602_)/(R_724_ + R_602_)	RhS	− 0.613**
NVI_724-719_	(R_724_-R_719_)/(R_724_ + R_719_)	RhS	− 0.718**
NVI_560-447_	(R_560_-R_447_)/(R_560_ + R_447_)	ScR	0.100
NVI_560-567_	(R_560_-R_567_)/(R_560_ + R_567_)	ScR	− 0.855**
NVI_560-579_	(R_560_-R_579_)/(R_560_ + R_579_)	ScR	− 0.840**
NVI_560-602_	(R_560_-R_602_)/(R_560_ + R_602_)	ScR	− 0.802**
NVI_579-447_	(R_579_-R_447_)/(R_579_ + R_447_)	ScR	0.337**
NVI_579-567_	(R_579_-R_567_)/(R_579_ + R_567_)	ScR	0.815**
NVI_636-733_	(R_636_-R_733_)/(R_636_ + R_733_)	ScR	0.836**
NVI_560-1908_	(R_560_-R_1908_)/(R_560_ + R_1908_)	ScS	− 0.172*
NVI_575-1908_	(R_575_-R_1908_)/(R_575_ + R_1908_)	ScS	− 0.081
NVI_601-1908_	(R_601_-R_1908_)/(R_601_ + R_1908_)	ScS	0.790**
NVI_656-667_	(R_656_-R_667_)/(R_656_ + R_667_)	ScS	0.367**
NVI_667-601_	(R_667_-R_601_)/(R_667_ + R_601_)	ScS	0.182**
SR_559-602_	R_559_/R_602_	RhS	− 0.788**
SR_602-559_	R_602_/R_559_	RhS	0.789**
SR_643-559_	R_643_/R_559_	RhS	0.692**
SR_703-724_	R_703_/R_724_	RhS	0.741**
SR_1377-664_	R_1377_/R_664_	RhS	− 0.683**
SR_560-579_	R_560_/R_579_	ScR	− 0.840**
SR_579-560_	R_579_/R_560_	ScR	0.840**
SR_636-733_	R_636_/R_733_	ScR	0.836**
SR_560/1135_	R_560_/R_1135_	ScS	0.515**
SR_575-560_	R_575_/R_560_	ScS	0.826**
SR_601-560_	R_601_/R_560_	ScS	0.790**
SR_656-667_	R_656_/R_667_	ScS	0.366**
SR_656-726_	R_656_/R_726_	ScS	0.794**

Spearman correlation coefficients between disease severities (Disease classes 0, 1, 2, 3) and the newly developed vegetation indices calculated from reflectance data recorded on rocket leaves inoculated with *Rhizoctonia solani* (RhS), *Sclerotium rolfsii* (ScR), and *Sclerotinia sclerotiorum* (ScS) (Exp_Gui).

*p > 0.05; **p < 0.01.

### Performances of VIs at both leaf and canopy scales

**Figure 4:**
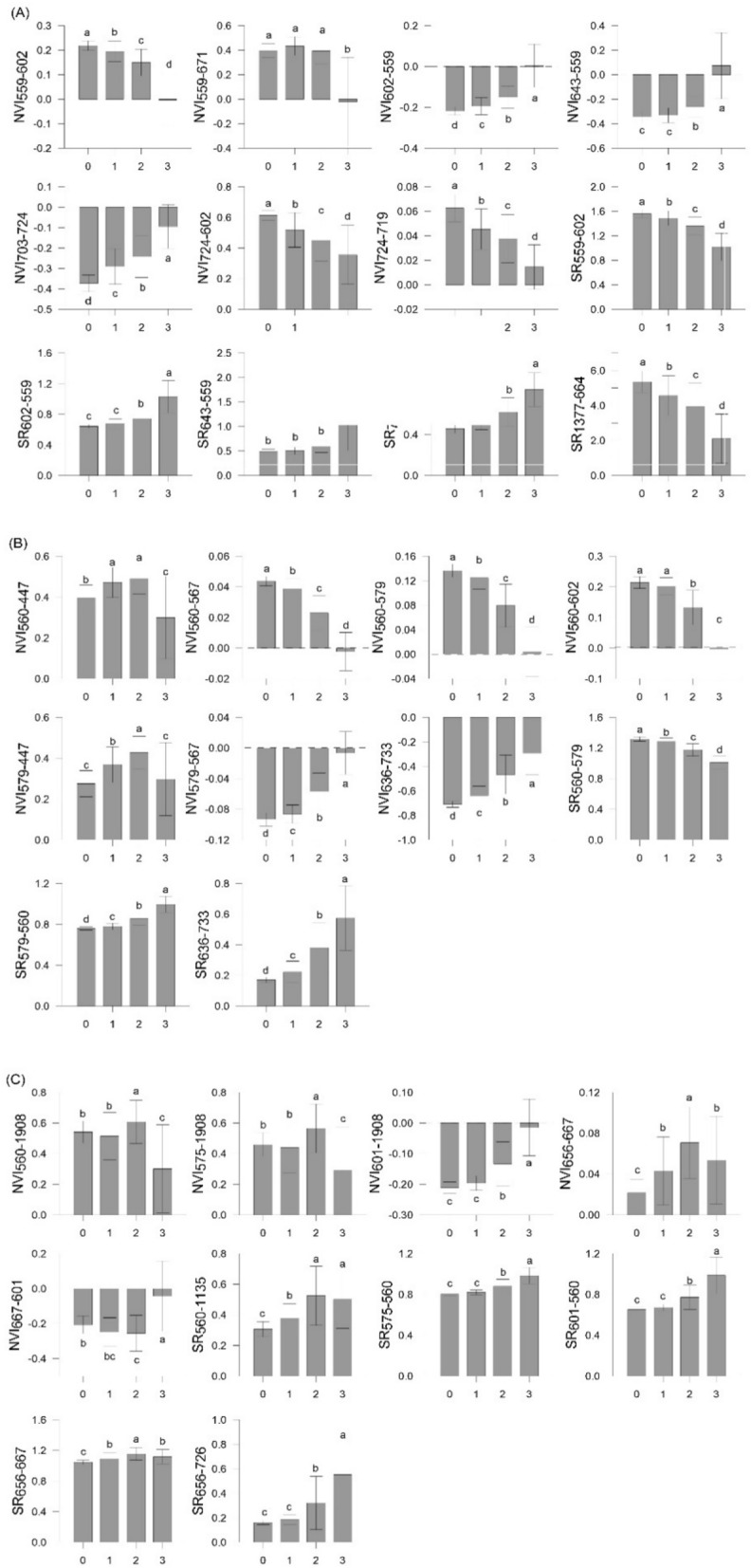
Average of the selected vegetation indices (VIs, dimensionless) calculated from the leaf-reflectance data of wild rocket inoculated with (**A**) *Rhizoctonia solani* (RhS-D), (**B**) *Sclerotium rolfsii* (ScR-D), and (**C**) *Sclerotinia sclerotiorum* (ScS-D) at four disease severity levels (Disease classes 0, 1, 2 and 3) (Exp_Gui). NVI = normalized differences index; SR = simple ratio. Bars with different lowercase letters are significantly different (*p*-value ≤ 0.05).

For each pathogen, the selected NVIs and SRs derived from Exp-Gui data are reported in Fig. 4. The highest number of new VIs was found for RhS-D, with 9 wavelengths falling within the ranges of green (559 nm), orange (602 nm), red (643, 664, 671, 703, 719, 724 nm) and SWIR (1377 nm) regions. Ten VIs (7 NVIs and 3 SRs) were obtained to discriminate ScR-D, based on 7 wavelengths of the VIS–NIR ranges, i.e., blue (447 nm), green (560 and 567 nm), yellow (579 nm), orange (602 nm), and red (636, and 733 nm). Lastly, 10 VIs were selected for ScS-D foliar symptoms discrimination: such VIs were developed starting from 8 wavelengths falling within green (560 nm), yellow (575 nm), orange (601 nm), red (656, 667, and 726 nm), and SWIR (1135 and 1908 nm) regions (Table 4). Some VIs were significantly different for all stages of pathogenesis (i.e., for RhS-D: NVI_559-602_, NVI_703-724_, NVI_724-602_, NVI_724-719_, SR_559-602_, SR_703-724_, and SR_1377-664_; for ScR-D: NVI_560-567_, NVI_560-579_, NVI_579-567,_ NVI_636-733_, SR_560-579_, and SR_636-733_), while others discriminated only the highest level of disease severity (i.e., for RhS-D: SR_643-559_; for ScS-D: SR_575-560_, and SR_601-560_) (Fig. 4).

Table 4 also displays the correlations among the selected VIs and the disease severity levels (Spearman correlation coefficient > 0.6 with *p*-value < 0.05). In general, high correlation coefficients were observed for RhS-D and ScR-D (except for NVI_559-671_ for RhS-D and NVI_560-447_ and NVI_579-447_ for ScR-D), while only 4 VIs showed high correlations (> 0.75) with the classes of severity in the case of ScS-D (i.e., NVI_601-1908_, SR_575-560_, SR_601-560_, and SR_656-726_). However, to evaluate the transferability of the selected VIs to high-throughput environments (i.e., canopy experiments), only the VIs showing Spearman correlation coefficients > 0.60 were considered.

**Figure 5:**
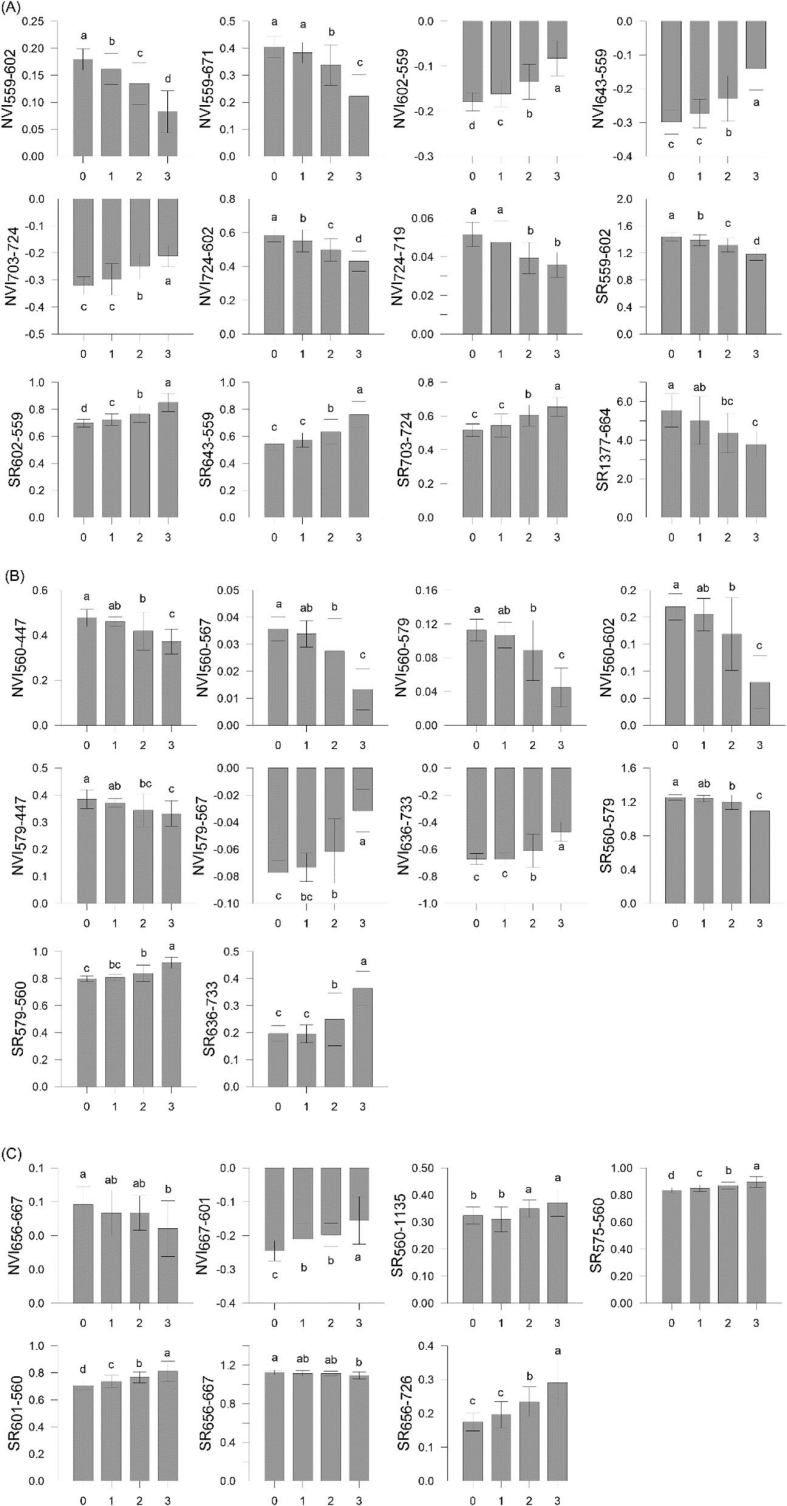
Average of the selected vegetation indices (VIs, dimensionless) calculated from the canopy reflectance data of wild rocket inoculated with (**A**) *Rhizoctonia solani* (RhS-D), (**B**) *Sclerotium rolfsii* (ScR-D), and (**C**) *Sclerotinia sclerotiorum* (ScS-D) at four disease severity levels (Disease classes 0, 1, 2 and 3) (Exp_App). NVI = normalized differences index; SR = simple ratio. Bars with different lowercase letters are significantly different (*p*-value ≤ 0.05).

The applicability of those VIs was tested using the canopy dataset from Exp_App. For each pathogen, VIs were calculated starting from canopy reflectance data at the pot scale (Fig. 5). Water absorption bands and noisy spectral regions over 1351–1409 nm and 1801–1949 nm were excluded from the canopy spectral signature and so the NVI_601-1908_ (ScS-D) was not calculated.

The identified VIs showed a significant relationship (based on Spearman correlation coefficients) with the disease classes of severity (Table 5). RhS-D maintained 11 VIs showing high correlation coefficients with disease levels (ranging from 0.60 to 0.65) but characterized by a good correlation (Pearson coefficient) with some important parameters of crop growth (i.e., LA and DM) (Table 5). The best results were achieved by the NVIs and SRs reporting the reflectance value at 559 and 602 nm. ScR-D presented the better performing indices among all the investigated pathogens. Those VIs were able to discriminate infection and well related to all measured growth parameters, including the plant DW at harvest. Finally, for ScS-D it was possible to maintain only three indices, characterized by a low correlation with disease severity, LA, and DM (Table 5).

**Table 5:** Spearman correlation coefficients for disease severities (Disease classes 0, 1, 2, 3) and Pearson correlation coefficients for dry weight (DW, g plot^−1^), leaf area (LA, cm^2^ plot^−1^), and dry matter (DM, %) and the newly developed vegetation indices (normalized difference indices: NVI; simple ratios: SR—listed in Table 4) calculated from reflectance data recorded on wild rocket inoculated with *Rhizoctonia solani* (RhS-D), *Sclerotium rolfsii* (ScR-D), and *Sclerotinia sclerotiorum* (ScS-D) (Exp_App).

Pathogen	Index	DI	DW	LA	DM
RhS-D	NVI_559-602_	− 0.650*	0.407*	0.756*	− 0.760*
	NVI_602-559_	0.650*	− 0.407*	− 0.756*	0.760*
	NVI_643-559_	0.627*	− 0.364*	− 0.743*	0.780*
	NVI_703-724_	0.629*	− 0.502*	− 0.738*	0.563*
	NVI_724-602_	− 0.625*	0.500*	0.745*	− 0.609*
	NVI_724-719_	− 0.604*	0.514*	0.669*	− 0.432*
	SR_559-602_	− 0.650*	0.408*	0.752*	− 0.732*
	SR_602-559_	0.650*	0.406*	− 0.756*	0.779*
	SR_643-559_	0.650*	0.365*	− 0.743*	0.807*
	SR_703-724_	0.627*	0.503*	− 0.748*	0.589*
	SR_1377-664_	− 0.629*	0.406*	0.585*	− 0.412*
ScR-D	NVI_560-567_	− 0.746*	0.654*	0.862*	− 0.799*
	NVI_560-579_	− 0.748*	0.672*	0.868*	− 0.801*
	NVI_560-602_	− 0.764*	0.675*	0.872*	− 0.815*
	NVI_579-567_	0.748*	− 0.676*	− 0.865*	0.795*
	NVI_636-733_	0.710*	− 0.674*ns*	− 0.844*	0.760*
	SR_560-579_	− 0.748*	0.668*	0.866*	− 0.791*
	SR_579-560_	0.748*	− 0.675*	− 0.869*	0.808*
	SR_636-733_	0.710*	− 0676*	− 0.841*	0.771*
ScS-D	SR_575-560_	0.590*	− 0.411*	− 0.625*	0.450*
	SR_601-560_	0.613*	− 0.404*	− 0.634*	0.494*
	SR_656-726_	0.607*	− 0.487*	− 0.659*	0.405*

Reflectance data were acquired at canopy scale (named as crop dataset; degrees of freedom = 78), so that was not possible to calculate all the newly developed vegetation indices (see the text for further information). Only indices showing significant  Spearman correlation coefficients for DI variable were reported.

*p > 0.05; *ns* non-significant.

## Discussion

Greenhouse cultivation of leafy vegetables such as wild rocket on large areas requires continuous disease monitoring for the ready identification of the spatial/temporal distribution of disease symptoms within the crop, which allows increasing effectiveness (less quantities) and targeting (more precision) of preventive and/or curative control means. For this purpose, proximal detection methods using optoelectronic probes have proved suitable to feed farmers’ decision-making processes by ensuring automatic, rapid, non-destructive, and large-scale disease assessment, and reducing the reaction time to the early outbreak^22^.

In this study, a high-resolution hyperspectral array [i.e., VIS/infrared (IR) spectroscopy] was used to attempt to follow the progression of *R. solani*, *S. rolfsii*, and *S. sclerotiorum* disease symptoms on *D. tenuifolia* through four different severity levels. To the best of our knowledge, this is the first study that investigates the responses of wild rocket to soil-borne fungal pathogens in terms of spectral reflectance. Starting from the full spectral signature we introduced and calculated new vegetation indices aimed at reducing the dimensionality of the dataset, suitable to discriminate between healthy and infected classes of wild rocket leaves, which could be effectively transferable to real (or real like) growing conditions, in order to accelerate disease detection. The methodological information obtained in the present study can be transferred to other (leaf) crop—(soil-borne) pathogen systems.

Modelling was performed on an experiment conducted under controlled conditions (i.e., growth chamber) and reflectance data were collected in the laboratory (at leaf scale) using a specific light source^41^, thus resulting in high-resolution and high-precision measurements. As expected, fungal diseases influenced the spectral signature: from a qualitative point of view, healthy plants showed low reflectance values in the VIS and SWIR regions as well as high reflectance values in the NIR, highlighting changes in physiological and biological parameters in responses to pathogenesis, i.e., reduction in chlorophyll content, changes in leaf water content, and degeneration of internal leaf structure, respectively^42–44^. In agreement with previous results^41^, a slight blue-shift in the position of the red-edge due to the symptoms of developing necrosis was also observed. However, slight differences among pathogens were detected (e.g., ScR-D near 1700 nm). This deserves further investigation as specific pathosystems are characterized by specific spatiotemporal dynamics, as reported in sugar beet^32^, tomato^45^, and potato^46^. It is worth specifying that the soil-borne diseases studied generally allow comparable symptomatology, despite differences in terms of disease progression, and their impact on foliage is almost indirect.

The latter aspect might also have been related to the ability of the RF models to discriminate healthy and highly infected leaves with higher accuracy, in comparison with intermediate disease severity, as previously observed in similar studies performed with Propagation Neural Network spectral calibration models on blackleg potato that reduced accuracy on whole plants in field trials^25^. The RF algorithm represents one of the most common statistical machine learning methods used in modelling and classifying qualitative classes of disease severity from hyperspectral data and has been successfully employed in many host–pathogen combinations^41,47^. Recently, the RF algorithm has been applied by our group to model the detection of powdery mildew and tracheofusariosis in wild rocket^48,49^. However, since the continuous narrowband datasets contain redundant spectral information, the selection of significant wavebands was performed, based on the results of RF decision trees^50^.

The main objective of this study was to obtain new spectral VIs, starting from the selected wavelengths in the three full-spectrum regions (i.e., VIS, NIR and SWIR), useful in the scaling up and speeding up of disease detection^51^ in wild rocket plants under greenhouse or open-field conditions. In conceptual agreement with the described pipeline, similar hyperspectral data mining was previously conducted to identify the best indices with high healthy/diseased separation capacity of late blight in tomato^52^, Cercospora leaf spot in beet^53^, powdery mildew in squash^29^, bacterial wilt in peanut^27^, Yellow Rust in wheat^54^, and Alternaria leaf blight in potato^55^.

Regardless of fungal diseases, the new calculated VIs (at leaf scale) were generally effective in separating healthy from infected leaves; however, some VIs could also discriminate intermediate disease intensities. Interestingly, in the case of ScR-D, which seemed to be characterized by the highest hyperspectral spectroscopy performances, probably due to its greater aggressiveness, the selected NVIs and SRs indices are based on green and light red bands highlighting the effects of disease on leaf pigments concentrations as well as on photosynthetic efficiency^56^. The SR_1377-664_ was characterized by a good ability to separate disease intensities associated with RhS-D, confirming the role of IR bands to estimate cell damages. In particular, the spectral region centered around 1377 nm represented a key region in the identification of physically damaged mushrooms during the monitoring of their quality^57^. Moreover, for RhS-D, the selected wavelengths in green and orange regions, at 559 and 602 nm, determined high-performance VIs. Those wavelengths have been found ascribed to the leaf chlorophyll content. In particular, λ559, which is normally ignored in most VIs, has been found to be associated with yield and biomass accumulation^58^.

To verify the transferability of de novo VIs, they were tested against the canopy dataset. Close to the spectral regions of the VIs in the present study, MCARI has been previously found to be responsive to the early stage of disease development of *Cercospora* leaf spot and rust on sugar beet^32^, to tomato chlorophyll content and LAI reduction as affected by bacterial wilting^59^, and to asymptomatic shoot status in presence of abiotic root stress in pepper^60^. On the other hand, the indices estimating pigments, Plant Pigment Ratio and Carotenoid Indices, based respectively on range 550–450 and 500–570 nm, have been, respectively, used to predict plant N status and chlorophyll^61^ and carotenoid levels as a proxy of plant stress^62^.

As a matter of fact, the ongoing symptoms associated with soil-borne pathogens, i.e., losses of chlorophyll and other pigments, discoloration, reduced water content, wilting and, finally, desiccation, are closely linked to reduced leaf absorbance in the canopy^63^. In sugar beet, Reynolds et al.^64^ calculated two narrowband hyperspectral vegetation indices, such as Pigment Specific Simple Ratio (working on chlorophyll a) and modified Spectral Ratio (close to canopy reflectance at 445, 705 and 750 nm), for early detection of *Rhizoctonia* crown and root rot, while chlorophyll/carotenoids dependent SRPI (simple ratio pigment index) was proposed to discriminate the soil borne disease from *Heterodera schachtii* nematode symptoms^65^. Huang and Apan^66^ found VIS–NIR narrow range wavelengths valuable to predict the incidence of *Sclerotinia* rot disease in celery. For most of the literature about VIs, the discrimination between healthy and severely diseased plants leaves is attributable to the strong changes that occurred in the later soil-borne infection stages^24^. The relevance of these indices seems to be limited to the drastic exclusion of infected plants that can be successfully practised, whereas, in the late stage, disease progression is too advanced to apply preventive strategies.

In the present study, the newly formulated VIs at high-resolution and low-throughput levels (leaf scale) were tested directly on an independent dataset (high-throughput experiment), built on reflectance measurements performed on the canopy, confirming their transferability to ordinary growing and measuring conditions. Interestingly, for all diseases a good correlation between computable VIs and LA was observed.

## Conclusions

Our study underlines the importance of scaling up the results of reflectance spectroscopy, performed at leaf scale with a contact probe, under controlled conditions and thus without by environmental factor effects (i.e., light intensity and direction, temperature, and canopy architecture), to larger-scale experiments (canopy scale), characterized by increasingly high-throughput and important for practical applications.

New vegetation indices able to discriminate disease stages were obtained. The most interesting wavelengths fell in the visible and infrared spectral regions, including interesting regions/wavelengths already investigated and previously identified under stress conditions. Extraction of the most significant spectral information from the big dataset is crucial to simplify the production of optoelectronic sensors, suitable for plant disease detection^41^ specifically aiming to cheap and innovative surveying devices, to support sustainable disease management in greenhouse or open-field conditions. On perspective, this technology, possibly integrated with different sensors (i.e., thermal sensors and/or 3-D shape sensors)^67^, can improve pathogen detection, since the availability of significant spectral information linked to machine vision-based remote sensing makes feasible the real-time mapping of disease management.

## Supplementary Information

Supplementary Information.
