# Supplementary material for: Surveying soil-borne disease development on wild rocket salad crop by proximal sensing based on high-resolution hyperspectral features
Source: Sci Rep. 2022 Mar 24;12:5098. doi: 10.1038/s41598-022-08969-5 (PMC8948195; doi:10.1038/s41598-022-08969-5)
Supplement: Supplementary file 1 — Supplementary Information. [file 41598_2022_8969_MOESM1_ESM.docx]

**Supplementary materials**

**
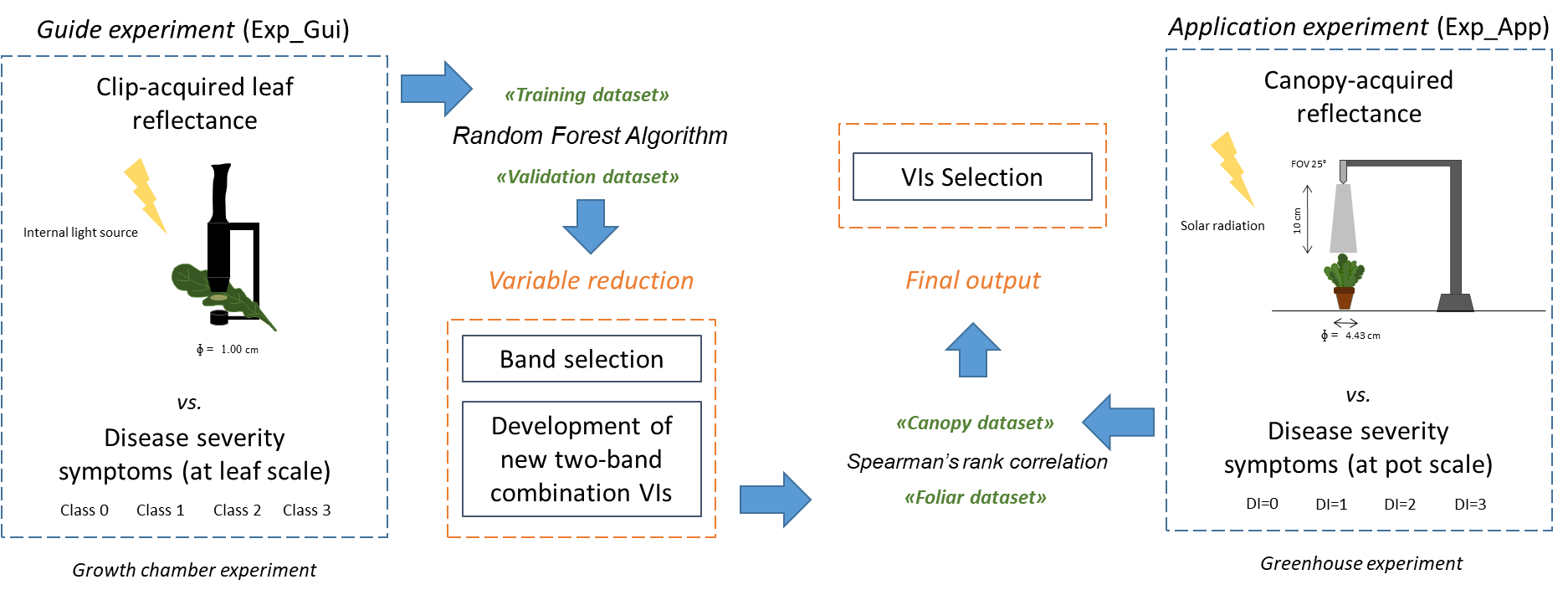
**

**Fig. S1.** Flowchart of sensitive band selection, new indices construction and testing for soil-borne disease detection in wild rocket.

**Table S1.** Statistics by Class of the Confusion Matrix assessing the prediction performances of Random Forest models trained and validated on all wavebands (All λ), selected wavebands (Selected λ), all VIs generated by the combinations of the selected wavelengths (All VIs), and only the selected VIs (selected VIs) using the *Rhizoctonia solani* disease (RhS-D) dataset (Exp_Gui). Sensitivity, true positives; Specificity, true negatives; Pos Pred Value, positives captured on the total of predicted as positives; Neg Pred Value, negatives captured on the total of predicted as negatives; Prevalence, all actual positives in the population; Detection, all the detected cases on the total; Detection Prevalence, all the predicted positives on the total; Balanced Accuracy, expressed as the average of Sensitivity and Specificity.

|  |  |  |  | **Statistics by Class** | | |  |  |  |
| --- | --- | --- | --- | --- | --- | --- | --- | --- | --- |
|  | Disease Class | Sensitivity | Specificity | Pos Pred  Value | Neg Pred  Value | Prevalence | Detection  Rate | Detection  Prevalence | Balanced  Accuracy |
| **All λ** | 0 | 0.8571 | 0.7600 | 0.5000 | 0.9500 | 0.2344 | 0.1875 | 0.3750 | 0.8086 |
|  | 1 | 0.5000 | 0.7727 | 0.5000 | 0.7727 | 0.2344 | 0.1562 | 0.3125 | 0.6364 |
|  | 2 | 0.2667 | 0.9592 | 0.6667 | 0.8103 | 0.3125 | 0.0625 | 0.0938 | 0.6129 |
|  | 3 | 0.9333 | 1.0000 | 1.0000 | 0.9800 | 0.2188 | 0.2188 | 0.2188 | 0.9667 |
| **Selected λ** | 0 | 0.9474 | 0.7955 | 0.6667 | 0.9722 | 0.3016 | 0.2857 | 0.4286 | 0.8714 |
|  | 1 | 0.5789 | 0.9318 | 0.7857 | 0.8367 | 0.3016 | 0.1746 | 0.2222 | 0.7554 |
|  | 2 | 0.7143 | 0.9592 | 0.8333 | 0.9216 | 0.2222 | 0.1587 | 0.1905 | 0.8367 |
|  | 3 | 0.8182 | 0.9808 | 0.9000 | 0.9623 | 0.1746 | 0.1429 | 0.1587 | 0.8995 |
| **All VIs** | 0 | 0.9286 | 0.7200 | 0.4815 | 0.9730 | 0.2188 | 0.2031 | 0.4219 | 0.8243 |
|  | 1 | 0.3500 | 0.9091 | 0.6364 | 0.7547 | 0.3125 | 0.1094 | 0.1719 | 0.6295 |
|  | 2 | 0.6000 | 0.9592 | 0.8182 | 0.8868 | 0.2344 | 0.1406 | 0.1719 | 0.7796 |
|  | 3 | 0.8667 | 0.9592 | 0.8667 | 0.9592 | 0.2344 | 0.2031 | 0.2344 | 0.9129 |
| **Selected VIs** | 0 | 0.7143 | 0.8000 | 0.5000 | 0.9091 | 0.2188 | 0.1562 | 0.3125 | 0.7571 |
|  | 1 | 0.3500 | 0.7955 | 0.4375 | 0.7292 | 0.3125 | 0.1094 | 0.2500 | 0.5727 |
|  | 2 | 0.3333 | 0.8163 | 0. 3571 | 0.8000 | 0.2344 | 0.0781 | 0.2188 | 0.5748 |
|  | 3 | 0.7333 | 0.9388 | 0.7857 | 0.9200 | 0.2344 | 0.1719 | 0.2188 | 0.8361 |

**Table S2.** Statistics by Class of the Confusion Matrix assessing the prediction performances of Random Forest models trained and validated on all wavebands (All λ), selected wavebands (Selected λ), all VIs generated by the combinations of the selected wavelengths (All VIs), and only the selected VIs (selected VIs) using the *Sclerotium rolfsii* disease (ScR-D) dataset (Exp_Gui). Sensitivity, true positives; Specificity, true negatives; Pos Pred Value, positives captured on the total of predicted as positives; Neg Pred Value, negatives captured on the total of predicted as negatives; Prevalence, all actual positives in the population; Detection, all the detected cases on the total; Detection Prevalence, all the predicted positives on the total; Balanced Accuracy, expressed as the average of Sensitivity and Specificity.

|  |  |  |  | **Statistics by Class** | | |  |  |  |
| --- | --- | --- | --- | --- | --- | --- | --- | --- | --- |
|  | Disease Class | Sensitivity | Specificity | Pos Pred  Value | Neg Pred  Value | Prevalence | Detection  Rate | Detection  Prevalence | Balanced  Accuracy |
| **All λ** | 0 | 0.8889 | 0.8636 | 0.7273 | 0.9500 | 0.2903 | 0.2581 | 0.3548 | 0.8763 |
|  | 1 | 0.6111 | 0.8409 | 0.6111 | 0.8409 | 0.2903 | 0.1774 | 0.2903 | 0.7260 |
|  | 2 | 0.5000 | 0.9565 | 0.8000 | 0.8462 | 0. 2581 | 0.1290 | 0.1613 | 0.7283 |
|  | 3 | 1.0000 | 0.9615 | 0.8333 | 1.0000 | 0.1613 | 0.1613 | 0.1935 | 0.9808 |
| **Selected λ** | 0 | 0.9444 | 0.7955 | 0.6538 | 0.9722 | 0.2903 | 0.2742 | 0.4194 | 0.8699 |
|  | 1 | 0.5000 | 0.8636 | 0.6000 | 0.8085 | 0.2903 | 0.1452 | 0.2419 | 0.6818 |
|  | 2 | 0. 4375 | 0.8913 | 0.5833 | 0.8200 | 0.2581 | 0.1129 | 0.1935 | 0.6644 |
|  | 3 | 0.8000 | 0.9808 | 0.8889 | 0.9623 | 0.1613 | 0.1290 | 0.1452 | 0.8904 |
| **All VIs** | 0 | 0.8333 | 0.7500 | 0.5769 | 0.9167 | 0.2903 | 0.2419 | 0.4194 | 0.7917 |
|  | 1 | 0.4444 | 0.8409 | 0.5333 | 0.7872 | 0.2903 | 0.1290 | 0.2419 | 0.6427 |
|  | 2 | 0.4375 | 0.9565 | 0.7778 | 0.8302 | 0.2581 | 0.1129 | 0.1452 | 0.6970 |
|  | 3 | 1.0000 | 0.9615 | 0.8333 | 1.0000 | 0.1613 | 0.1613 | 0.1935 | 0.9808 |
| **Selected VIs** | 0 | 0.7778 | 0.8409 | 0.6667 | 0.9024 | 0.2903 | 0.2258 | 0.3387 | 0.8093 |
|  | 1 | 0.5000 | 0.7727 | 0.4737 | 0.7907 | 0.2903 | 0.1452 | 0.3065 | 0.6364 |
|  | 2 | 0.4375 | 0.8913 | 0.5833 | 0.8200 | 0.2581 | 0.1129 | 0.1935 | 0.6644 |
|  | 3 | 0.8000 | 0.9615 | 0.8000 | 0.9615 | 0.1613 | 0.1290 | 0.1613 | 0.8808 |

**Table S3.** Statistics by Class of the Confusion Matrix assessing the prediction performances of Random Forest models trained and validated on all wavebands (All λ), selected wavebands (Selected λ), all VIs generated by the combinations of the selected wavelengths (All VIs), and only the selected VIs (selected VIs) using the *Sclerotinia sclerotiorum* disease (ScS-D) dataset (Exp_Gui). Sensitivity, true positives; Specificity, true negatives; Pos Pred Value, positives captured on the total of predicted as positives; Neg Pred Value, negatives captured on the total of predicted as negatives; Prevalence, all actual positives in the population; Detection, all the detected cases on the total; Detection Prevalence, all the predicted positives on the total; Balanced Accuracy, expressed as the average of Sensitivity and Specificity.

|  |  |  |  | **Statistics by Class** | | |  |  |  |
| --- | --- | --- | --- | --- | --- | --- | --- | --- | --- |
|  | Disease Class | Sensitivity | Specificity | Pos Pred  Value | Neg Pred  Value | Prevalence | Detection  Rate | Detection  Prevalence | Balanced  Accuracy |
| **All λ** | 0 | 0.8235 | 0.7143 | 0.5000 | 0.9211 | 0.2576 | 0.2121 | 0.4242 | 0.7689 |
|  | 1 | 0.1500 | 0.9130 | 0.4286 | 0.7119 | 0.3030 | 0.0455 | 0.1061 | 0.5315 |
|  | 2 | 0.4615 | 0.7925 | 0.3529 | 0.8571 | 0.1970 | 0.0909 | 0.2576 | 0.6270 |
|  | 3 | 0.6250 | 0.9200 | 0.7143 | 0.8846 | 0.2424 | 0.1515 | 0.2121 | 0.7725 |
| **Selected λ** | 0 | 0.8824 | 0.6939 | 0.5000 | 0.9444 | 0.2576 | 0.2273 | 0.4545 | 0.7881 |
|  | 1 | 0.2000 | 0.9565 | 0.6667 | 0.7333 | 0.3030 | 0.0606 | 0.0909 | 0.5783 |
|  | 2 | 0.5385 | 0.7925 | 0.3889 | 0.8750 | 0.1970 | 0.1061 | 0.2727 | 0.6655 |
|  | 3 | 0.6250 | 0.9600 | 0.8333 | 0.8889 | 0.2424 | 0.1515 | 0.1818 | 0.7925 |
| **All VIs** | 0 | 0.7647 | 0.7551 | 0.5200 | 0.9024 | 0.2576 | 0.1970 | 0.3788 | 0.7599 |
|  | 1 | 0.2500 | 0.8478 | 0.4167 | 0.7222 | 0.3030 | 0.0758 | 0.1818 | 0.5489 |
|  | 2 | 0.6154 | 0.8113 | 0.4444 | 0.8958 | 0.1970 | 0.1212 | 0.2727 | 0.7134 |
|  | 3 | 0.5625 | 0.9600 | 0.8182 | 0.8727 | 0.2424 | 0.1364 | 0.1667 | 0.7612 |
| **Selected VIs** | 0 | 0.8235 | 0.7347 | 0.5185 | 0.9231 | 0.2576 | 0.2121 | 0.4091 | 0.7791 |
|  | 1 | 0.2500 | 0.8913 | 0.5000 | 0.7321 | 0.3030 | 0.0758 | 0.1515 | 0.5707 |
|  | 2 | 0.5385 | 0.8302 | 0.4375 | 0.8800 | 0.1970 | 0.1061 | 0.2424 | 0.6843 |
|  | 3 | 0.6875 | 0.9600 | 0.8462 | 0.9057 | 0.2424 | 0.1667 | 0.1970 | 0.8237 |
